# An Integrative Computational Approach for Prioritization of Genomic Variants

**DOI:** 10.1371/journal.pone.0114903

**Published:** 2014-12-15

**Authors:** Inna Dubchak, Sandhya Balasubramanian, Sheng Wang, Cem Meyden, Dinanath Sulakhe, Alexander Poliakov, Daniela Börnigen, Bingqing Xie, Andrew Taylor, Jianzhu Ma, Alex R. Paciorkowski, Ghayda M. Mirzaa, Paul Dave, Gady Agam, Jinbo Xu, Lihadh Al-Gazali, Christopher E. Mason, M. Elizabeth Ross, Natalia Maltsev, T. Conrad Gilliam

**Affiliations:** 1 Genomics Division, Lawrence Berkeley National Laboratory, Berkeley, California, United States of America; 2 Department of Energy Joint Genome Institute, Walnut Creek, California, United States of America; 3 Department of Human Genetics, University of Chicago, Chicago, Illinois, United States of America; 4 Toyota Technological Institute at Chicago, Chicago, Illinois, United States of America; 5 Department of Physiology and Biophysics, Weill Cornell Medical College, New York, New York, United States of America; 6 The HRH Prince Alwaleed Bin Talal Bin Abdulaziz Alsaud Institute for Computational Biomedicine, Weill Cornell Medical College, New York, New York, United States of America; 7 Feil Family Brain and Mind Research Institute, Weill Cornell Medical College, New York, New York, United States of America; 8 Computation Institute, University of Chicago/Argonne National Laboratory, Chicago, Illinois, United States of America; 9 Department of Computer Science, Illinois Institute of Technology, Chicago, Illinois, United States of America; 10 Departments of Neurology, Pediatrics, and Biomedical Genetics and Center for Neural Development and Disease, University of Rochester Medical Center, Rochester, New York, United States of America; 11 Seattle Children's Research Institute and Department of Pediatrics, University of Washington, Seattle, Washington, United States of America; 12 Department of Pediatrics, Faculty of Medicine and Health Sciences, United Arab Emirates University, Al-Ain, UAE; 13 Laboratory of Neurogenetics and Development, Weill Cornell Medical College, New York, New York, United States of America; Central China Normal University, China

## Abstract

An essential step in the discovery of molecular mechanisms contributing to disease phenotypes and efficient experimental planning is the development of weighted hypotheses that estimate the functional effects of sequence variants discovered by high-throughput genomics. With the increasing specialization of the bioinformatics resources, creating analytical workflows that seamlessly integrate data and bioinformatics tools developed by multiple groups becomes inevitable. Here we present a case study of a use of the distributed analytical environment integrating four complementary specialized resources, namely the Lynx platform, VISTA RViewer, the Developmental Brain Disorders Database (DBDB), and the RaptorX server, for the identification of high-confidence candidate genes contributing to pathogenesis of spina bifida. The analysis resulted in prediction and validation of deleterious mutations in the SLC19A placental transporter in mothers of the affected children that causes narrowing of the outlet channel and therefore leads to the reduced folate permeation rate. The described approach also enabled correct identification of several genes, previously shown to contribute to pathogenesis of spina bifida, and suggestion of additional genes for experimental validations. The study demonstrates that the seamless integration of bioinformatics resources enables fast and efficient prioritization and characterization of genomic factors and molecular networks contributing to the phenotypes of interest.

## Introduction

The identification of genomic variations contributing to specific phenotypes of direct medical relevance is an ultimate goal of numerous studies in human genetics. The development of solid weighted hypotheses on the functional effects of a sequence variant is an essential step for gaining insights into a genetic architecture of a disease and for the efficient planning of experiments. However, as the volume and complexity of biological information increases, it demands sophisticated analytical workflows involving multitude of steps for extraction of actionable knowledge. In the past years much attention in the bioinformatics literature was given to data integration [1]–[3]. Seamless integration of complementary services and tools provided by multiple groups in workflow pipelines is essential for comprehensive data analysis. It provides the means for substantial reduction of time and effort required for analysis of translational data and significant increase in the efficiency of knowledge extraction.

A number of excellent bioinformatics platforms and tools have been developed in the recent years to support various steps of analysis of high-throughput data and prioritization of genomic variants (reviewed in [4]–[6]). These include, but not limited to GeneMANIA [7], STRING [8], [9], ToppGene [10], Endeavour [11] widely used by the scientific community. The eXtasy platform developed by Sifrim et al. [12] prioritizes mutations for follow-up validation studies by integrating variant-impact and haploinsufficiency predictions with phenotype-specific information. Another scientific environment, SPRING [13], has been designed to facilitate the prioritization of pathogenic non-synonymous SNVs associated with the disorders whose genetic bases are either partly known or completely unknown. It is achieved by integrating the results of analyses by multiple publicly available and developed in-house bioinformatics tools. There are more analytical platforms, such as Jannovar [14], KGGSeq [15], MToolBox [16] and FamAnn [17]. Moreover, multiple resources support the analysis of non-coding regions and their regulatory roles [18]. Most of these existing resources, understandably, address either the analysis of coding sequences or the characterization of non-coding regions.

**Figure 1:**
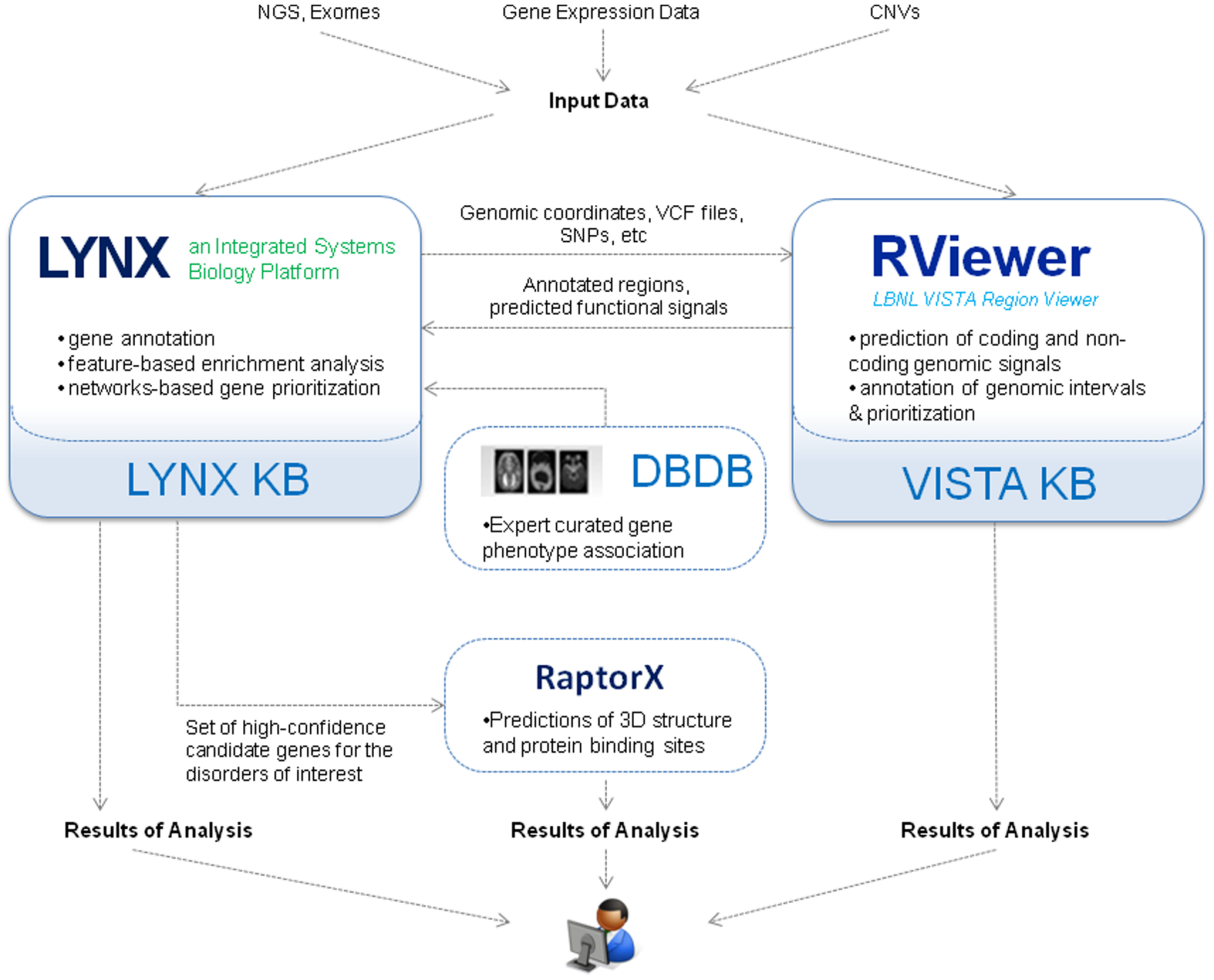
Integration of services in the described analytical environment. Lynx logo © 2013–2014 Department of Genetics, University of Chicago. RViewer logo © 2010–2012 The Regents of the University of California.

The analytical environment described here however is different from these resources. It is based on seamless integration of data and services across multiple independently developed analytical systems and databases, namely the Lynx [19] and the VISTA [20] systems, the Developmental Brain Disorders Database (DBDB) [21], and the RaptorX server [22], [23]. This environment, depicted in Fig. 1, allows end users to easily direct and analyze their data among all these systems. The benefits of such integration are manifold. They include the integration of the vast knowledge bases developed by each system to support the annotation of the experimental data and the subsequent analyses. Complementary analytical tools and the Web services-based collaborative interfaces provide flexible analytical pipelines seamlessly operating across the participating systems.

As an example, we have demonstrated an ability of the reported pipeline to identify polymorphisms that make plausible candidates for factors contributing to spina bifida (SB), using whole genome next generation sequence (NGS) data for affected patients and their parents. We show advantages of an integrated approach for both hypothesis-based and discovery-based methods for identification and prioritization of genetic factors contributing to complex developmental phenotypes. The presented example also serves as a proof of concept for the integration of various computational resources for the high-throughput analysis of genomic variants.

## Materials and Methods

### 1. Integrative Analytical Approach

We have integrated the following analytical resources developed by four groups: (1) VISTA RViewer [24] for the annotation and comparative and evolutionary analysis of coding and non-coding regions of the genomes; (2) the Lynx platform [19] supporting enrichment analysis and networks-based gene prioritization, (3) the Developmental Brain Disorders Database (DBDB) [21], and (4) RaptorX [23] for predicting 3D structure and functional properties of identified candidate gene products. Combining knowledge bases and knowledge-extraction services into a seamlessly integrated analytical pipeline creates a one-stop solution for generating weighted hypotheses regarding the molecular mechanisms contributing to the phenotypes of interest.

#### Data submission

The approach supports multiple entry points for annotation and analysis of translational data (e.g. genes, pathways, disorders), as well as batch queries via Web-based user interfaces or Web-services (see Fig. 1). The following queries can be submitted to Lynx or VISTA RViewer for annotation or downstream analysis (Fig. 1): (a) single gene queries to Lynx, RViewer or RaptorX; (b) search-based queries to Lynx or VISTA to retrieve information from these systems knowledge bases, (c) batch queries for experimental data in the form of SNPs, genomic coordinates, or gene lists. VISTA RViewer also supports analysis of the Variant Call Format (VCF) files. The results of gene expression analyses in a form of a table containing gene symbol – expression value pair may be directly uploaded into the Lynx Network-based prioritization engine. Lynx Web site provides access to detailed tutorials.

### 2. Participating Analytical Resources

The sections below will describe the components of the integrated distributed analytical pipeline in more details.

#### VISTA Region Viewer (RViewer)

VISTA RViewer [24], one of the VISTA comparative genomics programs [20] widely adopted by the biomedical community [25]–[27], employs a new concept of comparative analysis for automating the prioritization of functional variants based on comparative genomics. VISTA RViewer allows for comparison and prioritization of the entire functional content of several genomic regions in parallel. Examples of such uses could be sets of CNVs regions from experiments, genomic neighborhoods of SNPs from GWAS or other studies, genes of expression studies, etc. RViewer has several functions not found in other currently available tools [28]–[30], namely it enables the simultaneous comparison of functional features in multiple genomic intervals, i.e. provides capabilities for quicker analysis, prioritization and visual inspection. RViewer takes as input genetic variation data from different biomedical studies (e.g.: GWAS, exomes), and determines a number of functional parameters for both coding and noncoding sequences in each region. Each gene in the region is characterized using the following contexts: (a) biological function; (b) features of protein products of these genes; (c) tissue expression; (d) binding partners (e) developmental stage; (f) pathways and networks information from pathways databases and literature (g) known disease associations; and (h) known genetic variations. For noncoding regions, RViewer provides: homotypic clusters of transcription factor binding sites, a key component of promoters and enhancers [31]; experimentally verified enhancers from the VISTA Enhancer browser [32]; heart- and hindbrain- specific enhancers derived computationally [33], [34]; and conserved TFBS in the promoters of all human genes. In addition, a wide range of comparative genomics data based on pairwise and multiple alignments [35] is accessible.

In the LYNX - VISTA integrative system, RViewer plays a dual role, both calculating a number of functional parameters used by LYNX in further analysis, and visualizing a set of genomic regions with prioritized variant provided to a user as an output of the system. In particular, it finds a genomic position of a submitted by a user variant (coordinate and a specific position in intron, exon, UTRs, intergenic), associates it with UCSC isoforms, calculates deleteriousness of the non-synonymous coding SNPs using Polyphen2 [36], and defines occurrence of SNPs in clusters of TFBS [31], enhancers [32], and highly conserved intervals in inter-species pairwise and multiple alignments. Prioritized genes and other functional features in resulting genomic intervals are displayed in RViewer with all relevant functional content for further interactive exploration by a user.

#### Lynx annotation and knowledge extraction engine

Lynx (http://lynx.ci.uchicago.edu) is an integrated bioinformatics platform and a knowledge extraction engine for annotation and analysis of high-throughput biomedical data [19]. Lynx receives user data as genomic variants whose coding and non-coding signals have been characterized by RViewer (Fig. 1). The platform supports both hypothesis-based and discovery-based approaches to prediction of genetic factors and networks associated with phenotypes of interest. It provides a knowledge extraction engine and a supporting knowledge base (LynxKB) combining various classes of information from over thirty-five public databases and private collections. Lynx knowledge retrieval engine offers advanced search capabilities and a variety of algorithms for gene enrichment analysis and network-based gene prioritization. Lynx's XML schema-driven annotation service supports extraction of annotations for an individual object (e.g. a gene) or batch queries (e.g. list of genes) from LynxKB. Annotations include *inter alia* associated pathways, diseases, phenotypes, molecular interactions, Gene Ontology categories, toxicogenomic information displayed according to the user preferences. All information related to the objects is easily accessible via user interface and available for download in tab-delimited, XML or JSON formats (Web Services).

Lynx gene enrichment analysis supports Bayes factor and p-value estimates for identification of functional categories over-represented in the query data sets (see B. Xie et al. for more details [37]). Lynx enrichment analysis is based on a large variety of features (e.g. Gene Ontology terms, toxicogenomic information, tissues), as well as unique for the system customized brain connectivity ontology, symptoms-level phenotypes and associated non-coding signals from VISTA (e.g. enhancers and clusters of transcription factors binding sites). Lynx also supports context-sensitive enrichment analysis (e.g. against genes expressed on a particular developmental stage or in a particular tissue) that may substantially increase the accuracy of the results.

Additionally, Lynx integrates five network propagation algorithms (simple random walk, heat kernel diffusion, PageRank with priors, HITS with priors and K-step Markov) as initially developed in the gene prioritization tool PINTA [38]. These algorithms were modified for Lynx to replace continuous gene expression data with binary data from seed genes. This modification accommodates the use of a variety of weighted data types for gene prioritization including ranked gene to phenotype associations, weighted canonical pathways, gene expression, results of sequencing analyses and others. STRING v 9.0 [8] is used as the underlying protein interaction network. Networks-based gene prioritization facilitates prioritization of promising candidate genes from large gene sets or even from the entire genome to provide a preliminary step for network reconstruction. Lynx Service Oriented Architecture provides public access to LynxKB and its analytical tools via user-friendly web services and interfaces.

*Developmental Brain Disorders Database (DBDB).* In a lot of cases the analysis of translational data requires integration of the domain- and project specific data (e.g. phenotypic and clinical data, tissue-specific information). Significant amount of this information is already integrated into the Lynx knowledge base. However, the described analytical pipeline can be customized to integrate additional information resources (e.g. public databases and private collections) to meet the user requirements. Here we present the integration of the information from the Developmental Brain Disorders Database (DBDB) [39] as an example of satisfying the needs of neurodevelopmental research.

DBDB (https://www.dbdb.urmc.rochester.edu/home) is a publicly available, curated on-line repository of genes, phenotypes, and syndromes associated with human neurodevelopmental disorders [21]. The discovery of genetic mechanisms contributing to pathogenesis of diseases relies on pre-existing knowledge regarding complex phenotype-genotype relationships accumulated in the libraries of disease candidate genes (e. g. OMIM [40], AutDB [41]), databases describing genetic variations (e.g. GAD [42], SLEP [43]) or journal publications. The accuracy of these data varies significantly and affects the quality of the developed models. DBDB addresses this issue by employing a novel system for estimating levels of evidence for over 850 gene-phenotype associations curated by domain knowledge experts. While a useful tool for clinical diagnostics, DBDB is also increasingly valuable for annotation of variants identified from next-generation sequencing experiments and the development of predictive computational models for the disorders of interest. Here, DBDB was used for assigning of the levels of confidence to the gene-phenotype associations used for the reconstruction of molecular pathways potentially contributing to pathogenesis of Spina bifida.

#### RaptorX

RaptorX (http://raptorx.uchicago.edu) [23] is a protein structure and function prediction web server, excelling at predicting 3D structures for protein sequences without close homologs in the Protein Data Bank (PDB). Given a query sequence, RaptorX predicts its secondary and tertiary structures as well as solvent accessibility and disordered regions. RaptorX also predicts the binding sites of a protein sequence, based upon the predicted 3D model.

RaptorX applies a template-based approach to protein structure prediction. To deal with cases where no close template exists, RaptorX employs a couple of novel modeling strategies. First, RaptorX integrates a variety of context-specific biological signals in a non-linear probabilistic scoring function by using a powerful machine learning model Conditional Neural Fields (CNF) [44]. Second, RaptorX uses a multiple-template threading (MTT) procedure to significantly improve both alignment and modeling accuracy for some targets with multiple similar templates [45]. The predictions and tertiary structure models produced by RaptorX can serve as starting points for further analysis in a number of diverse application areas. For example, the predicted 3D models used for binding site prediction can also be used for epitope prediction as well as in protein-protein interaction studies. Given a protein sequence, RaptorX predicts its binding sites by aligning its predicted 3D model to a database of ligand-binding protein structures using a structure alignment tool DeepAlign [46].

### 3. Integrated Environment

The systems described above provide complementary services for identification and characterization of causative factors and molecular mechanisms associated with phenotypes of interest. While they all act as individual entry points for the end users to start the analysis, the implemented integrative tools provide an environment for seamless data transfers between the systems. Following are a few examples of the interfaces built towards integrating these systems:

- **Lynx and RViewer.** After entering a list of genes, genetic variants (SNPs) or regions (CNVs) in the Lynx system, the users are provided with the interface to submit these datasets to RViewer for the annotations of the genomic regions including variants, transcription factor binding sites, experimental and computational enhancers and many others. The annotations data for transcription factor binding sites and enhancers is periodically fetched from the RViewer and integrated into the Lynx knowledge base and is used for the enrichment analysis of the genes of interest.
- **Lynx and DBDB.** The richly curated data in DBDB make possible providing weights to the genes of interest for particular phenotypes. These data can be used in Lynx's networks based prioritization interface. DBDB also provides direct links to the Lynx's single gene and multi-gene annotation pages.
- **Lynx and RaptorX.** Lynx provides a novel interface for submission of protein sequences directly to RaptorX, thus creating capabilities for RaptorX to predict the structure and binding site for high-confidence genes flawlessly.

## Results and Discussion

### Use Case: Identification of genetic factors contributing to the pathogenesis of spina bifida from whole genome sequencing data using coding and non-coding variant analysis

Spina bifida is a common congenital birth defect with an average worldwide prevalence of one case per 2000 births. It manifests itself as failed closure of the embryonic neural tube [47], [48] resulting in a breach of several vertebrae leaving spinal cord and/or spinal nerves exposed or covered by only a thin membrane. To date the genetic mechanisms underlying this disorder still remain elusive, but many studies indicate the convergence of gene-gene and gene-environment interactions to produce the defect [49], [50].

**Figure 2:**
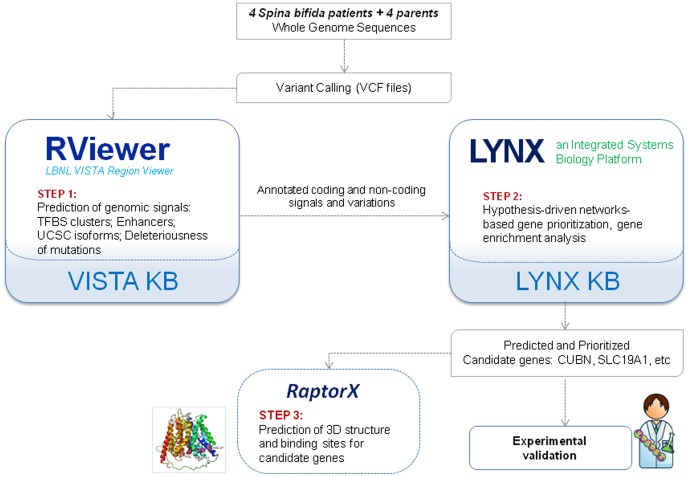
Analytical workflow for identification of spina bifida candidate genes.

In our study the Laboratory of Neurogenetics and Development (Weil Cornell Medical College) performed the whole genome sequencing for four patients with spina bifida, and four parents with normal phenotype from the same consanguineous family. The resulting SNPs were analyzed using both discovery-based, and hypothesis-based approaches for identification of the genetic factors contributing to the birth defect phenotype. The analysis included the following steps (schematically shown in Fig. 2):

- **Step 1**. *Discovery-based approach: annotation of genomic variants using VISTA RViewer.* SNPs were filtered based on quality and annotated using VISTA RViewer [24] with the following information: SNP to gene association (UCSC isoforms), deleteriousness of the non-synonymous SNPs using Polyphen2 [36], occurrence of SNPs in clusters of TFBS [31] and enhancers [32], genomic features associated with the region of the variant (intron, exon, UTRs). The Minor Allele Frequency (MAF) was estimated using ANNOVAR [51]. The resulting annotated SNPs were then filtered based on homozygosity, Minor Allele Frequency (MAF), and deleteriousness of mutations in exonic regions. All of the variants that were homozygous in probands but heterozygous in parents were extracted and used for further analysis to identify of de novo mutations in proband but not in the parents. A total of 743 variants in proband genomes were identified, out of which 6 variants were in the exonic region, 10 variants fell into UTRs, 337 variants were in the introns and the rest of the variants were in the intergenic un-annotated regions of the genome.
- **Step 2**. *Hypothesis driven approach to SNPs prioritization*: Defects in folate metabolism and transport were suggested to be contributing factors in pathogenesis of this birth defect [52], [53] however the exact genes associated with this disorder are not known. Here, thirty-nine genes involved in folate metabolism and transport were extracted from the literature [54], [55] and used as seed genes for network-based gene prioritization as implemented in Lynx [38]. STRING 9.0 [8] was used as a global probabilistic network. Network predicted genes with significant p-values <0.005 were used for subsequent analysis.

*Evaluation of the results*. Direct overlap with the known folate metabolism genes as well as the results of the network-based gene prioritization yielded a number of genes with rare variants (MAF = 0) in the exonic and UTR regions of both the patients and unaffected parents genes (Table 1 and Table S1 in S1 Materials).

**Table 1:** Identified genetic variants in folate metabolism genes in spina bifida patients and unaffected parents.

Gene	NW P-value*	Identified Variations	Variation Type	Variation occurrence in patients**	Variation occurrence in parents	Reference of association with SB
FOLH1	0	rs61886492	Missense, possibly damaging	6C1, 6C2		Morin, Devlin et al. 2003 [58]
DMGDH	0	rs532964	Missense, possibly damaging	2C1, 2C2, 6C1, 6C2	2M, 2F, 6M, 6F	Marini, Hoffmann et al. 2011 [53]
MTR	0	rs1805087	Missense, possibly damaging	2C1, 2C2, 6C1	2M, 2F, 6M, 6F	Doolin, Barbaux et al. 2002 [59]
MSGN1	0.002	rs35858730	Missense, possibly damaging	2C1, 2C2, 6C1, 6C2		Chalamalasetty, Dunty et al. 2011 [66]
CUBN	0.002	rs1801228	Benign		2M, 6M	Kozyraki, Fyfe et al. 1999, Wahlstedt-Froberg, Pettersson et al. 2003, Whitehead, 2006 [64], [68], [69]
		rs41289311				
SLC19A	0.0028	rs1051266	Missense, possibly damaging		2M, 2F, 6M, 6F	Shaw, Lammer et al. 2002, Morin, Devlin et al. 2003 [58], [67]
		rs2239911, rs2239908, rs2239907		2C1, 2C2, 6C2		

*The P-values in Table 1 are generated by 10 000 random permutations of the input data scored according to the strength of association with the phenotype using DBDB recommendations (random reassignment of the scores to network nodes and computation of the corresponding randomized scores for all candidate genes) [38].

**Family 1: affected children 2C1, 2C2; mother 2M, father 2F. Family 2: affected children 6C1, 6C2; mother 6M, father 6F.

In the recent years the genetic variations in genes involved in the folate-homocysteine metabolism were assumed to be possible risk factors for spina bifida [56]–[58]. These studies, however, are problematic due to the complexity of epistatic relationships between the affected genes and potential involvement of both the maternal and the offspring genotype in determining the pathogenic effect of these mutations.

As seen in Table 1, a number of patient genes involved in folate biosynthesis contained deleterious mutations. These included folate hydrolase 1 (FOLH1), mesagenin (MSGN1) and solute carrier family 19 folate transporter, member 1 (SLC19A1). The mesagenin 1 (MSGN1 gene) is known to be involved in specification of the paraxial mesoderm and regulation of the expression of T-box transcription factors required for mesoderm formation and differentiation. The mouse mutant of this gene is implicated in spina bifida phenotype. It was also suggested [59] that MSGN1 serves as one of the Wnt target genes in Wnt/β-catenin signaling that plays a well-established role in the regulation of embryonic and adult stem cell homeostasis. Folate hydrolase 1 (FOLH1), also found to contain deleterious mutations in two patients is known to act as a glutamate carboxypeptidase on different substrates, including the nutrient folate and the neuropeptide N-acetyl-l-aspartyl-l-glutamate [60].

Our analysis also identified a number of parental genes containing deleterious mutations. With respect to offspring, the effects of maternal genetic mutations may be considered to be environmental risk factors. Identified parental genes potentially contributing to spina bifida in affected children included CUBN1, MTR, SLC19A and DMGDH genes. Some of these genes were previously found to be associated with spina bifida. Doolin et al. [61], have demonstrated that methionine synthase (MTR) variants influence the risk of spina bifida via the maternal rather than the embryonic genotype. Moreover, in our study both mothers also showed an exonic variant (rs1051266) in the SLC19A1 gene encoding placental solute carrier family 19 folate transporter, member 1 (RFC) as it was demonstrated by RViewer. Polymorphisms in vitamin B receptor (CUBN) and in SLC19A1 (RFC) in mothers identified by networks-based gene prioritization have been previously shown to be associated with spina bifida or other neural tube defects in offspring [62]–[64] supporting the above inferences obtained in the course of our analysis.

#### Analysis and validation of the functional impact of the SLC19A1 mutation

**Figure 3:**
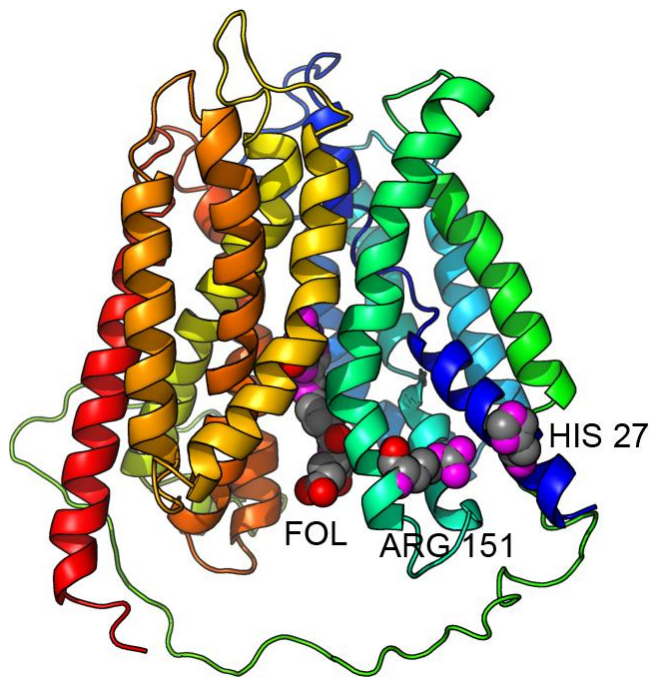
Ribbon diagram of SLC19A1 protein model generated by RaptorX. Rainbow coloring from blue to red indicates the N- to C-terminal positions of the residues in the model. The docking location of Folic Acid (FOL), shown in a spacefill form, was predicted by RaptorX-Binding. Numbers in black correspond to key residues, shown in spacefill form, which related to the functional impact of an exonic variant (rs1051266; Arg27His, 80G>A). The diagram was generated using PyMOL.

The known average distribution of the exonic variant (rs1051266; Arg27His, 80G>A) in SLC19A1 placental folate transporter in general population is 30∶25∶45 [65]. Further investigation of a potential impact of this variation on function was done using RaptorX. Fig. 3 presents the results of the predictions of SLC19A1 3D structure and the locations of the binding sites by RaptorX.

The reconstruction has shown that SLC19A1 protein has a typical MFS membrane transporter architecture with N- and C-terminus domains containing six trans membrane helices each [66]. It was previously demonstrated [67] that the RFC1 80A-allele is associated with reduced plasma folate. This phenomena could be explained by the reconstructed model in the following way: the 80G>A substitution leads to a change in position 27 on TM1 from histidine (80A allele) that has a relatively medium volume and neutral charge with Arg27 (80G allele) that has a larger volume and positive charge. A variation is located on the transmembrane helix (TM1) close to the intracellular outlet site. Such substitution would likely lead to the narrowing of the outlet channel due to the repulsion of Arg151 on TM5 due to electrostatic repulsion force and therefore to the reduced folate permeation rate (see Fig. 3).

**Figure 4:**
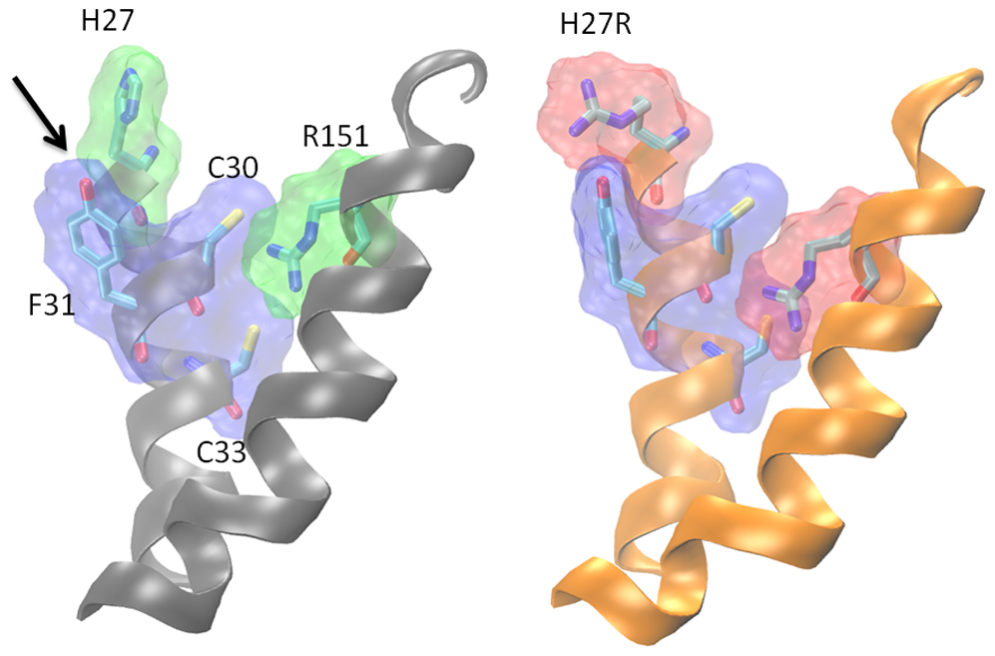
The changes to the binding site caused by the mutation (left: native, right: H27R mutant). The mutation results in changing contact landscape inside the cleft, especially for the Arg151 residue.

Indeed, an *in-silico* simulation and energy minimization study of the mutation (see Text S1 in S1 Materials for more details) shows that the area surrounding Arg151 becomes more compact and its contacting residues change after the mutation (see Fig. 4, and Figures S1 and S2 in S1 Materials). This results in 33% decrease in the volume of the cleft (from 807 Å^3^ to 541 Å^3^) and 28% decrease in the surface area of the cleft (from 442 Å^2^ to 328 Å^2^) (see Table 2 for details).

**Table 2:** Volume and surface area of the of the cleft in the native and the mutant structures as calculated by 3V.

Cleft Volume	Native	H27R Mutant
Volume	807 Å^3^	541 Å^3^
Surface area	442 Å^2^	328 Å^2^
Sphericity	0.95	0.98
Effective radius	5.47 Å	4.94 Å

**Figure 5:**
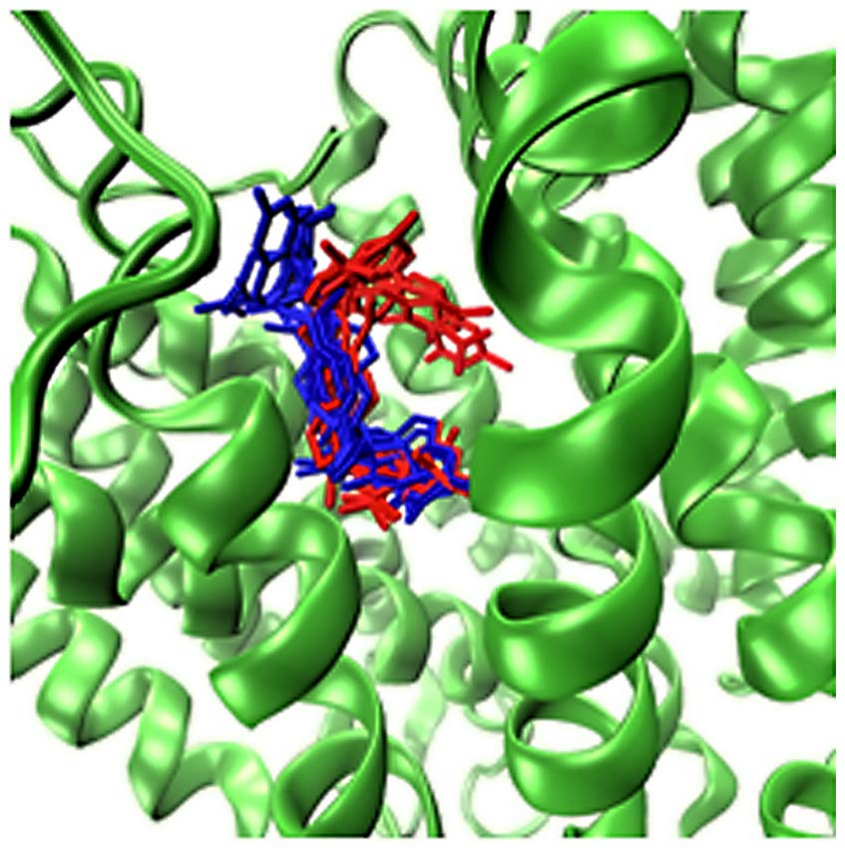
Docked folate conformations (blue: native, red: H27R mutant-bound folate molecules) showing the distinct change in the optimal conformation between native and the mutant.

Furthermore, a Pro-kink at Pro146 forces the side-chain of Phe141 to turn, narrowing the channel in front of the cleft (Figures S1 and S2 in S1 Materials). Further docking studies confirm that this narrowing results in a different conformation of binding for the folate (see Fig. 5 and Figures S3 and S4 in S1 Materials).

Annotation of SLC19A1 using Lynx showed that the expression and functionality of this gene is negatively affected by a number of pharmacological compounds, such as indomethacin, phenobarbital, nicotine and vitamin E. Analysis of potential use of these medications by mothers of affected children during pregnancy may provide additional clues regarding high occurrence of *spina bifida* in the consanguineous family under study.

The described approach has let us to correctly identify CUBN and SLC19A1 genes previously shown to contribute to pathogenesis of spina bifida [62]–[64] and to suggest additional genes for the next round of experimental validations.

#### Conclusions

The presented approach is an example of multilevel integration of the bioinformatics resources that offers seamless access to the knowledge bases, analytical tools and user interfaces independently developed by participating groups. Such integration is critically important for the progress of the translational studies since it significantly reduces the time and effort required to efficiently extract knowledge from the exponentially growing data sets produced by numerous genomics projects.

**Figure 6:**
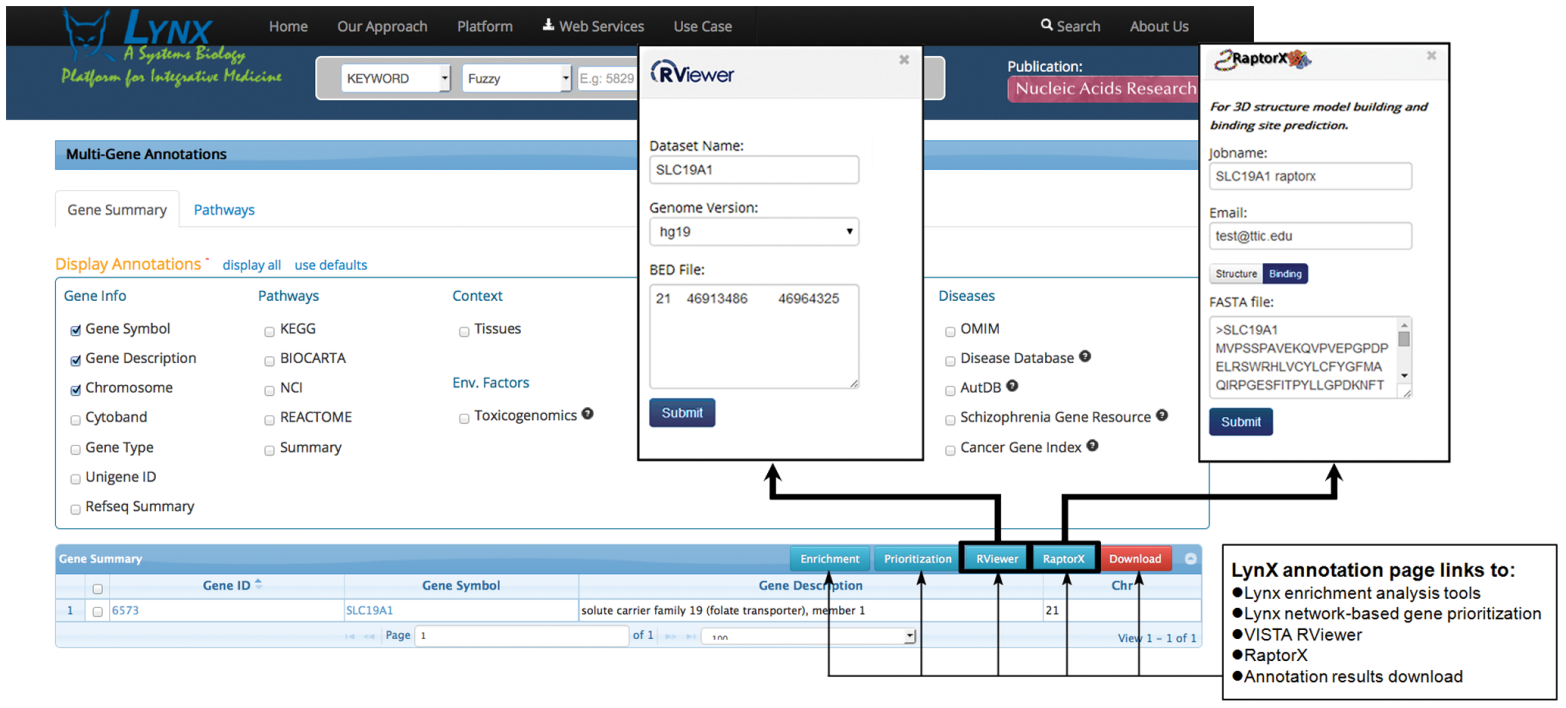
Access to the analytical tools within the described bioinformatics environment.

The spina bifida example demonstrates one of the possible analytical scenarios supported by the described computational framework. The presented here integrative approach however, can be generalized to support prioritization of the high-throughput experimental results and prediction of novel candidate genetic factors for any disorder of interest to the user. The power of the approach lies in massive integration of various classes of data from the Lynx (e.g. functional, phenotypic, pathways information), VISTA (e.g. genomic, evolutionary information), and RaptorX (proteomic and structural information). In the spina bifida example, the DBDB knowledge base was used as a source of domain-specific neurodevelopmental data. The resulting combined knowledge base may be used for extensive annotation of data and the results of analyses by these multiple resources. Another advantage offered by the described platform is the seamless integration of tools that supports the workflows spanning across the contributing resources (see Fig. 6). Datasets provided by the user in the form of SNPs, gene lists, genomic coordinates, VCF files, results of gene expression experiments, or obtained via queries to the participating knowledge bases may be analyzed by a variety of tools used in combinations suitable for the goals of a particular experiment.

The following types of analysis are currently integrated: RViewer provides an extensive annotation of genomic intervals of interest (e.g. known variations, TFBS); Lynx enrichment analysis allows the user to identify biological functions and phenotypes over-represented in the user datasets; Lynx network-based gene prioritization predicts high-confidence genes contributing to disease phenotypes; RaptorX server gives predictions of the 3D protein structure for protein sequences without close homologs in PDB, solvent accessibility, and disordered regions thus facilitating understanding of protein-ligand interactions.

We are working on the expansion of the array of available analytical services integrated in the described environment, specifically to provide access to additional domain-specific resources (e.g. cancer and cardiovascular studies). As the volume and complexity of biological information continues to increase, the seamless integration of bioinformatics platforms will offer a practical solution for the needs of biomedical studies.

## Supporting Information

Materials S1 **Supporting text, figures and table.** Text S1. Methodology. **Figure S1.** The overall view of the energy-minimized structures of SLC19A1, native (a) and H27R mutant (b). **Figure S2.** The changes to the binding site caused by the mutation (left: native, right: H27R mutant). (**a and b**) The mutation results in changing contact landscape inside the cleft, especially for the Arg151 residue. (**c**) Shift of Pro146 causes a kink in the loop, causing the helix to kink and the sidechain of Phe141 to turn, reducing the size of the cleft entrance. **Figure S3.** Visualization of the cleft volume and shape in both the native (left) and the mutant (right) structures. The H27R mutation reduces the volume of the cleft by 33%. **Table S1.** Volume and surface area of the cleft in the native and the mutant structures as calculated by 3V (ref).
(DOCX)
