# Correction: An Integrative Computational Approach for Prioritization of Genomic Variants

**DOI:** 10.1371/journal.pone.0124700

**Published:** 2015-04-08

**Authors:** The PLOS ONE Staff

The fourth author’s name is spelled incorrectly. The correct name is: Cem Meydan. The correct citation is: Dubchak I, Balasubramanian S, Wang S, Meydan C, Sulakhe D, Poliakov A, et al. (2014) An Integrative Computational Approach for Prioritization of Genomic Variants. PLoS ONE 9(12): e114903. doi:10.1371/journal.pone.0114903
