# Supplementary material for: An Integrative Computational Approach for Prioritization of Genomic Variants
Source: PLoS One. 2014 Dec 15;9(12):e114903. doi: 10.1371/journal.pone.0114903 (PMC4266634; doi:10.1371/journal.pone.0114903)
Supplement: Materials S1 — Supporting text, figures and table. Text S1. Methodology. Figure S1. The overall view of the energy-minimized structures of SLC19A1, native (a) and H27R mutant (b). Figure S2. The changes to the binding site caused by the mutation (left: native, right: H27R mutant). (a and b) The mutation results in changing contact landscape inside the cleft, especially for the Arg151 residue. (c) Shift of Pro146 causes a kink in the loop, causing the helix to kink and the sidechain of Phe141 to turn, reducing the size of the cleft entrance. Figure S3. Visualization of the cleft volume and shape in both the native (left) and the mutant (right) structures. The H27R mutation reduces the volume of the cleft by 33%. Table S1. Volume and surface area of the cleft in the native and the mutant structures as calculated by 3V (ref). (DOCX) [file pone.0114903.s001.docx]

**Text S1.**

**Methodology**

***In-silico* mutagenesis and energy minimization for the folate transporter SLC19A1.** The native structure and coordinates for SLC19A1 were obtained from RaptorX [[1](#_ENREF_1)]. Both the native structure and the H27R mutant systems were optimized by performing 10,000 steps of steepest descent and conjugate gradient in NAMD [[2](#_ENREF_2)] to eliminate improper contacts. VMD was used for visualization of structures [[3](#_ENREF_3),[4](#_ENREF_4)].

The channel finder algorithm in the 3V webserver [[5](#_ENREF_5)] was used to determine the binding cleft, its volume and surface in both the native structure and the H27R mutant.

**Molecular docking.** During docking, the energy minimized structures of SLC19A1 were taken as the receptor and the folic acid structure was taken as the ligand. The initial poses for the carrier-folic acid complex were determined using AutoDock Vina, which was selected since it is both fast and accurate, especially for relatively large ligands with many rotatable bonds such as folic acid [[6](#_ENREF_6)]. Autodock Vina predicts the dominant complexes of proteins and ligands with known 3D structures by decomposing their interaction energy into electrostatic, Lennard-Jones, hydrogen bonding, torsional, and solvation energies. The method calculates these scores over a pre-defined grid that includes the binding cleft [[7](#_ENREF_7)].

During the docking process folic acid possessed a torsion tree of 9 rotatable bonds and was treated as flexible while the receptor was kept rigid. The complex configurations obtained from docking were analyzed according to the poses and their scores.

**Results.** The structure after minimization is given in figures S1 and S2. Figure S2 shows in detail the changes to the proximity of the two alpha helices and to the sidechain configuration of Phe31. The mutation causes Arg151 of the binding cleft to come into contact with Cys30 and Cys33, reducing the cleft volume. Figure S3c also shows the kink at Pro146, causing the sidechain of Phe141 to rotate, narrowing the cleft entrance.

The change of size in the volume of the cleft was quantified using the 3V server [[5](#_ENREF_5)]. The H27R mutation results in a significant narrowing of the cleft, with a 33% decrease in the volume and a 28% decrease in the surface area (Table T1 and Figure S3).

The effects of the mutation to the folate binding were explored by docking the ligand to the native and mutant structures. Figure S4a visualizes the overlay of the top 9 ligand conformations for both structures, clearly showing the distinct profiles of folate binding for the native and the mutant. Root mean square distance (RMSD) calculation between all pairwise ligand modes (Figure S4b) further shows that the native and mutant bound ligands mostly cluster within themselves.

These results show that the H27R mutation is strongly predicted to alter the folate binding and transfer rate for the SLC19A1 protein, which confirms the clinical findings that correlate 80A (27R) allele with reduced plasma folate levels [[8](#_ENREF_8)]. However, study of folate transporter SLC19A1 is complicated by the lack of an experimentally determined high-resolution structure, which makes it infeasible to run molecular dynamics studies. Exploring the complex dynamics of membrane transport proteins would require experimentally validated protein-folate complex structures instead of predicted models. In that light, further studies might be necessary to find the exact change in the folate transport mechanism caused by different alleles in the population.

| **a-)** | 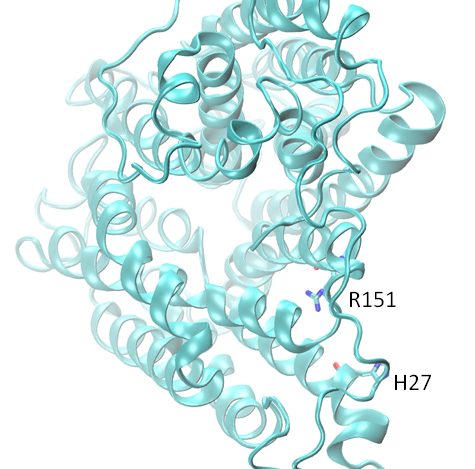 |
| --- | --- |
| **b-)** | 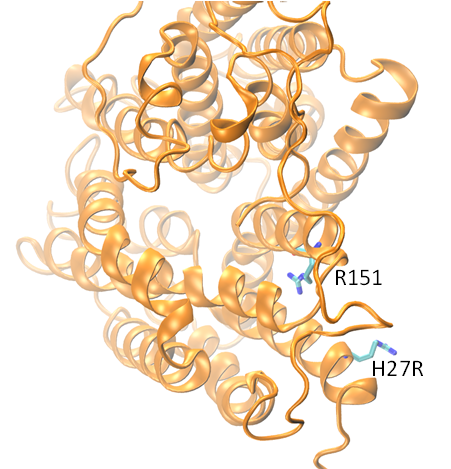 |
| **Figure S1.** The overall view of the energy-minimized structures of SLC19A1, native (a) and H27R mutant (b). | |

| **a-)** | **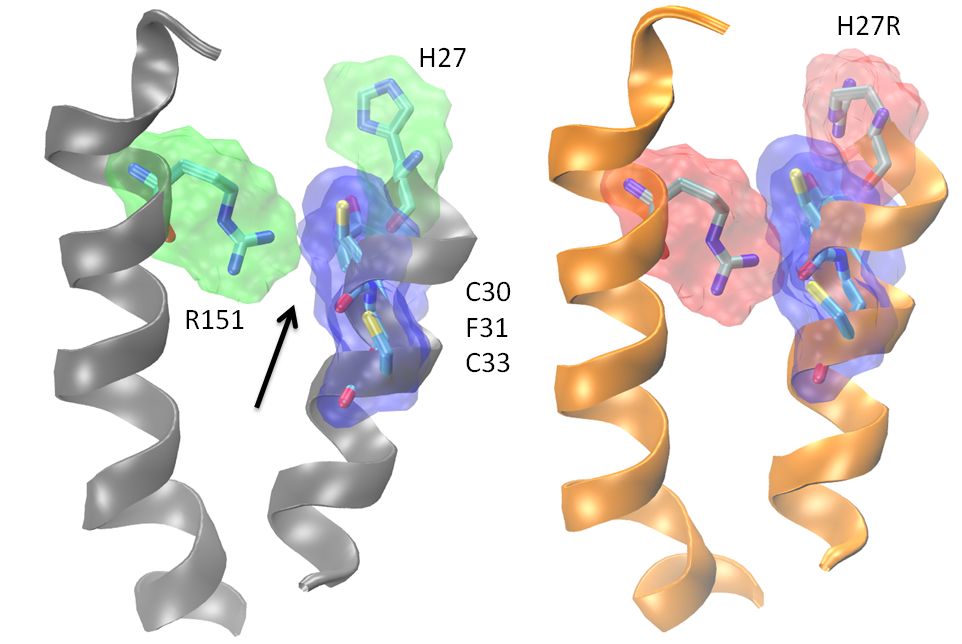** |
| --- | --- |
| **b-)** | 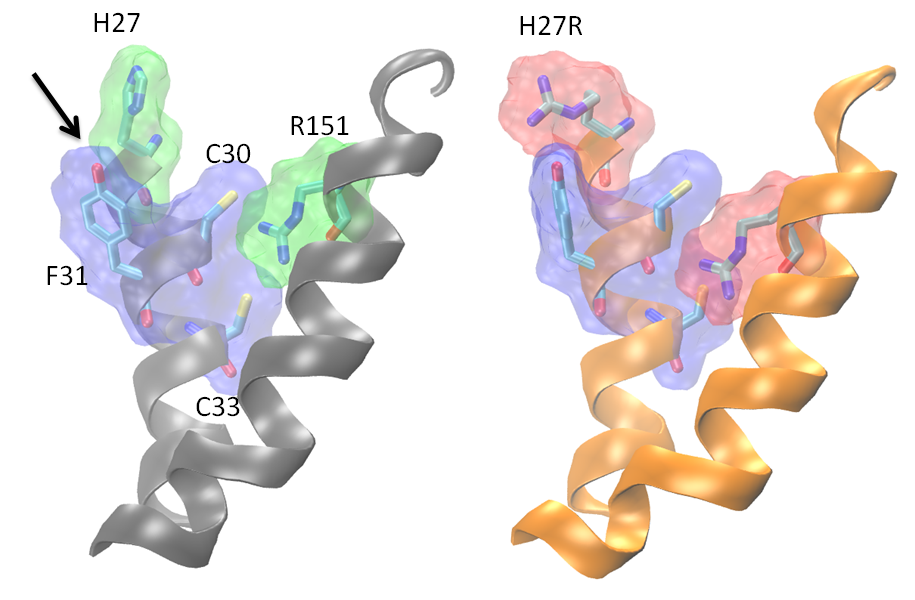 |
| **c-)** | **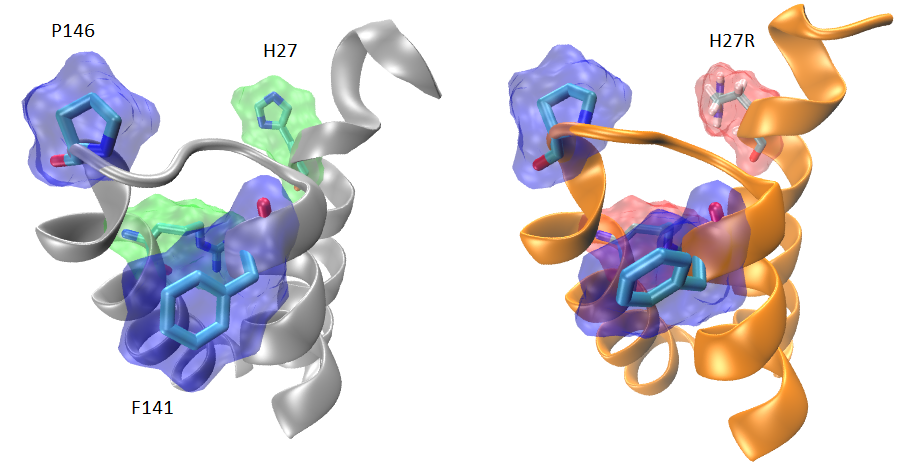** |
| **Figure S2.** The changes to the binding site caused by the mutation (left: native, right: H27R mutant). **(a and b)** The mutation results in changing contact landscape inside the cleft, especially for the Arg151 residue. **(c)** Shift of Pro146 causes a kink in the loop, causing the helix to kink and the sidechain of Phe141 to turn, reducing the size of the cleft entrance. | |

**Table S1.** Volume and surface area of the cleft in the native and the mutant structures as calculated by 3V (ref).

| Cleft Volume | Native | H27R Mutant |
| --- | --- | --- |
| Volume | 807 Å^3^ | 541 Å^3^ |
| Surface area | 442 Å^2^ | 328 Å^2^ |
| Sphericity | 0.95 | 0.98 |
| Effective radius | 5.47 Å | 4.94 Å |

| **Native Structure** | **H27R Mutant Structure** |
| --- | --- |
| 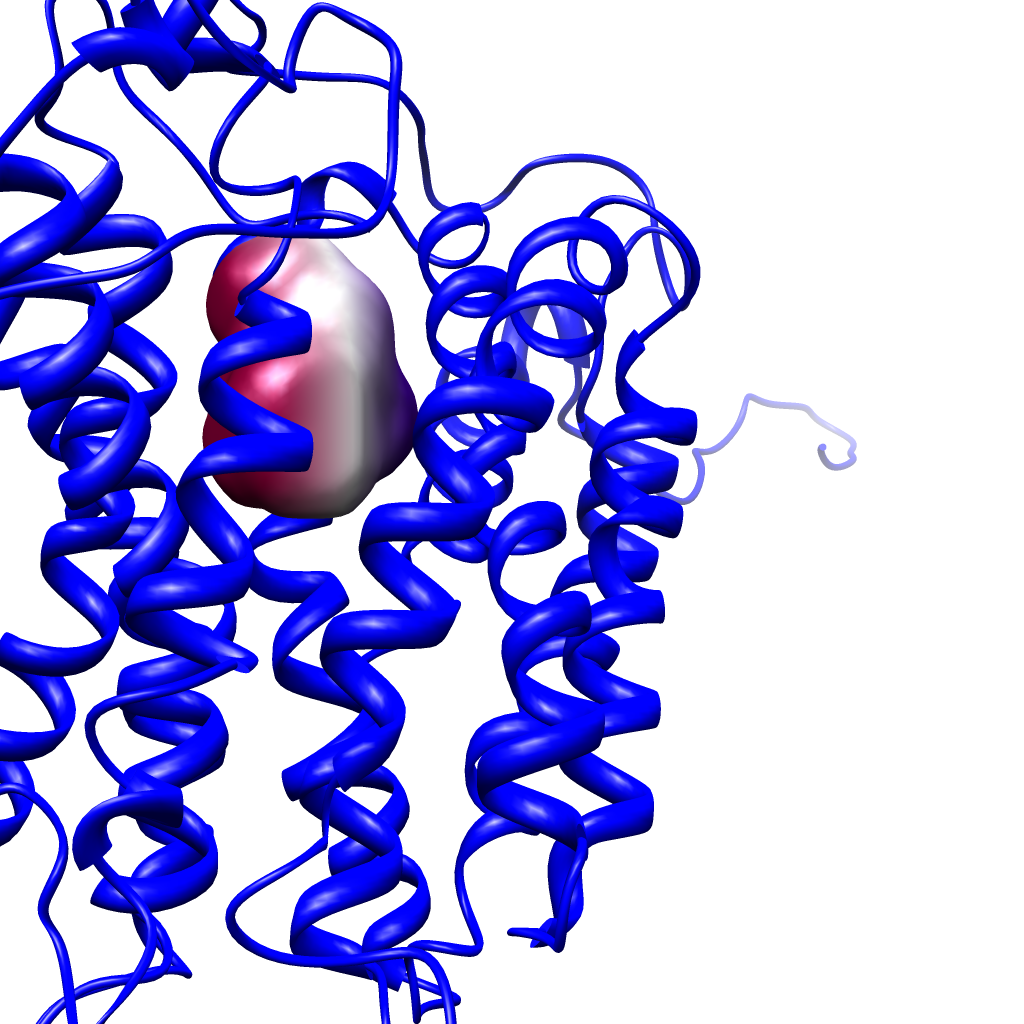 | 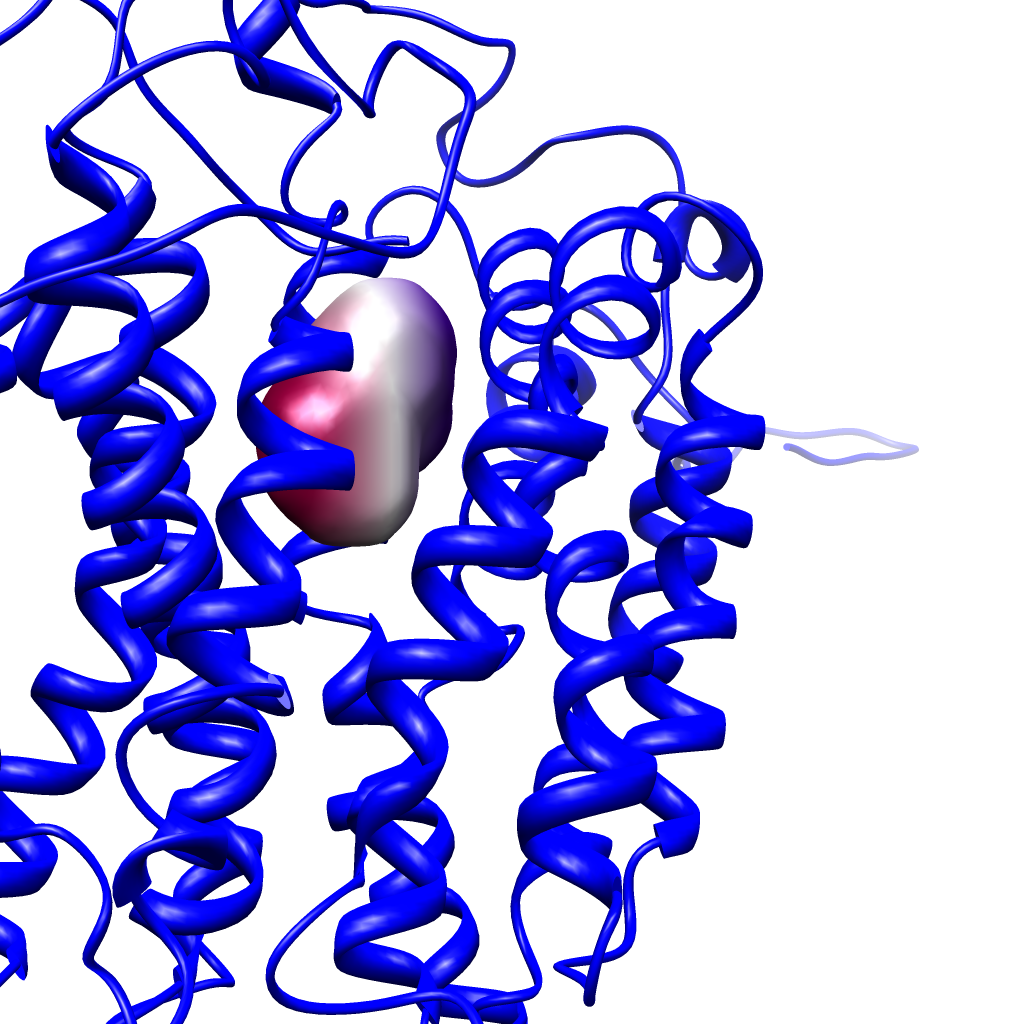 |
| **Figure S3.** Visualization of the cleft volume and shape in both the native (left) and the mutant (right) structures. The H27R mutation reduces the volume of the cleft by 33%. | |

| **a-)** | 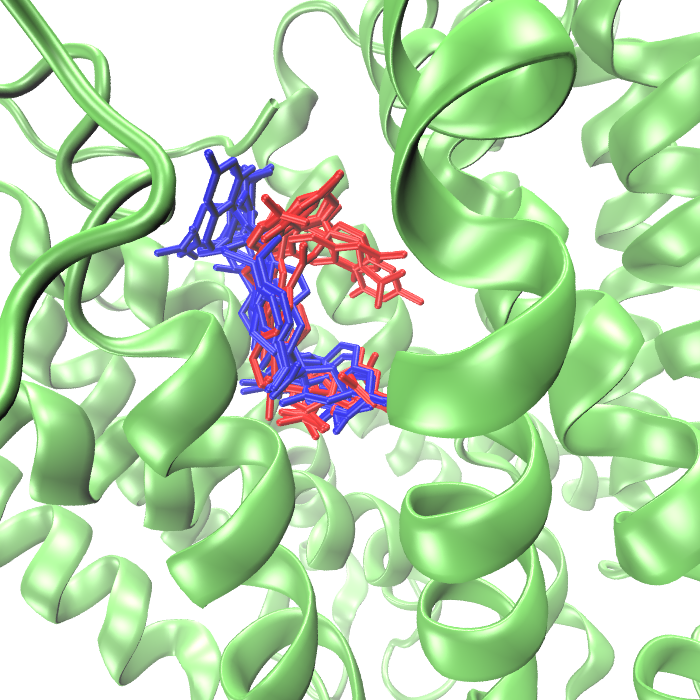 |
| --- | --- |
| **b-)** | 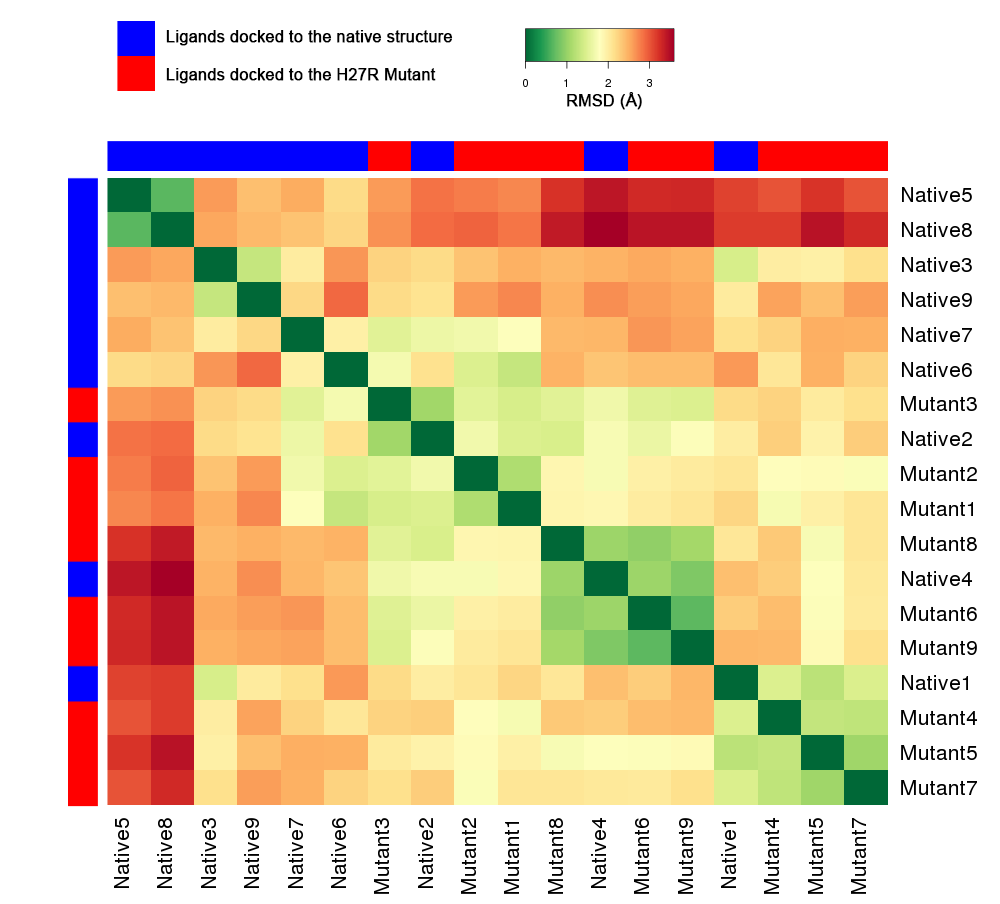 |
| **Figure S4.** **(a)** Docked folate conformations (blue: native, red: H27R mutant-bound folate molecules) showing the distinct change in the optimal conformation between native and the mutant. **(b)** Heatmap of the RMSD values between the different folate conformations. The two classes of docked ligands cluster mostly with other ligands from the same class (hierarchical Ward clustering). | |

**References**

1. Kallberg M, Wang HP, Wang S, Peng J, Wang ZY, et al. (2012) Template-based protein structure modeling using the RaptorX web server. Nature Protocols 7: 1511-1522.

2. Phillips JC, Braun R, Wang W, Gumbart J, Tajkhorshid E, et al. (2005) Scalable molecular dynamics with NAMD. J Comput Chem 26: 1781-1802.

3. Feig M, MacKerell J, A.D., Brooks I, C.L. (2003) Force field influence on the observation of a-helical protein structures in molecular dynamics simulations. Journal of Physical Chemistry B 107: 2831-2836.

4. MacKerell J, A.D. , Feig M, Brooks CL (2004) Improved treatment of the protein backbone in empirical force fields. Journal of the American Chemical Society 126: 698-699.

5. Voss NR, Gerstein M (2010) 3V: cavity, channel and cleft volume calculator and extractor. Nucleic Acids Res 38: W555-562.

6. Trott O, Olson AJ (2010) AutoDock Vina: improving the speed and accuracy of docking with a new scoring function, efficient optimization, and multithreading. Journal of Computational Chemistry 31: 455-461.

7. Morris GM, Goodsell DS, Huey R, Olson AJ (1996) Distributed automated docking of flexible ligands to proteins: parallel applications of AutoDock 2.4. J Comput Aided Mol Des 10: 293-304.

8. Stanislawska-Sachadyn A, Mitchell LE, Woodside JV, Buckley PT, Kealey C, et al. (2009) The reduced folate carrier (SLC19A1) c.80G>A polymorphism is associated with red cell folate concentrations among women. Ann Hum Genet 73: 484-491.
